# Nutrition and Osteoporosis: Preliminary data of Campania Region of European PERsonalised ICT Supported Service for Independent Living and Active Ageing

**Published:** 2016-01-31

**Authors:** L. Vuolo, L. Barrea, MC Savanelli, S. Savastano, M. Rubino, E. Scarano, M. Soprano, M. Illario, A. Colao, C. Di Somma

**Affiliations:** 1 Dipartimento di Medicina Clinica e Chirurgia, Sezione di Endocrinologia, Federico II University, Naples, Italy; 2 IOS & Coleman S.r.l, Naples; Italy.; 3 Department of Translational Medical Sciences, Federico II University, and R&D Unit, Federico II University Hospital; 4 IRCCS SDN Napoli

**Keywords:** nutrition, osteoporosis, ageing, Mediterranean diet

## Abstract

**Background:**

Bone impairment and malnutrition are associated with significant disability and mortality. PERSSILAA is an European project developing health services to detect and prevent frailty in older adults by addressing cognitive, physical and nutritional.

**Methods:**

Subjects underwent anthropometric measurements, calcaneal quantitative ultrasound (QUS) scan and PREDIMED (PREvención con DIeta MEDiterránea) questionnaire.

**Aim:**

To investigate the association between adherence to the Mediterranean Diet (MD) and bone health.

**Results:**

87 subjects (4 males and 83 females) 70.1±4.9 aged, were examined. Mean Body Mass Index (BMI) was 28.7±4.7(kg/m^2^): in particular 28 subjects (32.2%) resulted obese, 42 (48.3%) overweight, and only 17 (19.5%) with normal weight. Mean T score was −1.2±1.2: in particular 13 subjects (14.9%) resulted osteoporotic; 43 (49.5%) osteopenic; and 31 (35.6%) with normal bone mineral density. Regarding adherence to MD, 9 subjects (10.3%) were poorly adherent; 41 (47.2%) average adherent; 37 (42.5%) highly adherent. T-score was associated with PREDIMED score and osteoporotic subjects presented the lowest PREDIMED score (5.8±2.2).

**Conclusions:**

These preliminary data show a significant correlation between the adherence to the MD and bone health parameters. The association between MD and bone health highlights the potential beneficial effects of nutritional interventions promoting a Mediterranean food pattern, as safe adjuvant treatment in ageing.

## I. INTRODUCTION

Demographic ageing is a global trend. In the European Union, the number of people aged 65+ will almost double over the next 50 years, from 85 million in 2008 to 151 million in 2060. Among older adults, frailty is highly prevalent and constitutes a major health problem. Santos and Eggiman estimated the prevalence of frailty and pre-frailty in 10 European countries and showed that for those aged > 65 years frailty ranges between 5–27.3% and pre frailty between 35–51% of the population [1]. Frailty syndrome is a complex interaction between several factors, including but not limited to natural physiological alterations seen in aging, co- morbid diseases, nutritive and nutritional insufficiencies, cumulative negative environmental impact, genetics, and lifestyle choices. Frail individuals are vulnerable and at high risk of adverse health outcomes. They have functional impairments which often result in falls, immobility and confusion. Frailty limits regular physical activity and its many health benefits, including the prevention of cognitive decline. People affected by frailty make the most use of community resources, hospitals and long-term care institutions. Early screening for risk of frailty and functional decline in older adults and training of those who start to deploy frail conditions is key to successful prevention. Therefore it is important to screen for early detection of frailty in an integrated way as the cluster of clinical manifestations is probably at greater risk for adverse outcomes than any single component [2]. In this context PERsonalised ICT Supported Services for Independent Living and Active Ageing (PERSSILAA), a FP7 funded European project, develops and validates a new service model, to screen for and prevent frailty in community dwelling older adults, integrating nutrition, physical and cognitive function [3].

This multimodal service model, focusing on nutrition, physical and cognitive function, is supported by an interoperable ICT service infrastructure, utilising intelligent decision support systems and gamification. PERSSILAA, offered to older adults (> 65 years) through local community service, will be seamlessly integrated with health care services. PERSSILAA builds on activities within European Innovation Partnership on Active and Healthy Aging and on results of various earlier European projects. There is continuous end user involvement and evaluation with total 350 older adults in real implementation environments in Enschede (the Netherlands) and Campania Region (Italy) to ensure increasing system efficiency and easy end user acceptance [3]. Since lifestyle and diet impact on osteoporosis that is characterized by an increased risk for frailty fracture, disability, loss of independence, and even death, we focus on investigating the association between adherence to Mediterranean Diet (MD) and bone health status. In particular, the aim of our paper was to evaluate the association between anthropometric measurements, bone parameters and adherence to MD in community dwelling older adults (age>65 years), enrolled in Campania Region at local catholic churches involved in the first screening of PERSSILAA project.

## II. METHODOLOGY

PERSSILAA is an European project developing health services to detect and prevent frailty in older adults by addressing cognitive, physical and nutritional domains in Campania Region, Italy and Enschede, the Netherlands [3]. This community based healthcare service has been provided in Campania Region at local communities (catholic churches), in collaboration with the Department of Gastroenterology, Endocrinology and Surgery of Federico II University Hospital (Italy). The work has been carried out in accordance with the Code of Ethics of the World Medical Association (Declaration of Helsinki), and it has been approved by the Ethical Committee of the University of Naples “Federico II” Medical School. The purpose of the protocol was explained to all older adults enrolled, and written informed consent was obtained.

The study has been conducted on 87 adult patients from December 2014 to May 2015 including community dwelling older adults (age>65 years), enrolled in Campania Region at local catholic churches involved in the first screening of PERSSILAA project. Exclusion criteria were: Evident frailty, dependency in activities of daily living, immobility, moderate to advanced dementia. Anthropometric measurements, Adherence to MD and bone health status have been assessed in all subjects involved in the project.

All anthropometric measurements were taken with subjects wearing only light clothes and without shoes. In each subject, weight and height were measured to calculate the body mass index (BMI) [weight (kg) divided by height squared (m^2^), kg/m^2^]. Height was measured to the nearest 1 cm using a wall-mounted stadiometer. Body weight was determined to the nearest 50 g using a calibrated balance beam scale. Waist circumference (WC) was measured to the closest 0.1 cm using a non-stretchable measuring tape at the natural indentation or at a midway level between lower edge of the rib cage and iliac crest if no natural indentation was visible. The measurement was made with the subject standing upright, feet together and arms hanging freely at the sides, without compression of the soft tissue. According to the National Cholesterol Education Program’s Adult Treatment Panel III (NCEP-ATP III) criteria, abdominal obesity was defined as WC ≥102 cm in men and ≥88 cm in women [4]. Hip circumference was measured as the maximum circumference around the buttocks posteriorly and the symphysis pubis anteriorly, and measured to the nearest 0.5 cm. Waist-to-hip ratio (WHR) was calculated as the waist divided by the hip circumference.

A validated 14-item questionnaire for the assessment of adherence to the Mediterranean Diet, PREDIMED (PREvención con DIeta MEDiterránea) [5], was recorded for all the enrolled subjects during a face-to-face interview between the patient and a certified nutritionist or an endocrinologist. Briefly, for each item was assigned score 1 and 0; PREDIMED score was calculated as follows: score 0–5, low adherence; score 6–9, average adherence; score ≥10, high adherence [5].

Quantitative ultrasound (QUS) of the mid-calcaneus was performed with the Sahara Clinical Sonometer (Hologic, Bedford, MA, USA). The first US parameters employed for characterizing bone tissues are: Speed of Sound (SoS) and Broadband Ultrasound Attenuation (BUA). More complex parameters have been evaluated from combination of SoS and BUA: amplitude dependent speed of sound (AD-SoS), stiffness, quantitative ultrasound index (QUI); and then derived BMD, T score, and Z score. Calibration was performed before daily measurement according to the manufacturer’s instructions.

Results are expressed as mean ± SD or as median plus range according to variable distributions evaluated by Kolmogorov-Smirnov test. The chi 2 (χ^2^) test was used to test the significance of differences in frequency distribution. The correlations among variables were performed using Pearson *r* or Spearman’s *rho* correlation coefficients. In these analyses, we entered only those variables that had a *p*-value <0.05. To avoid multicollinearity, variables with a variance inflation factor (VIP) >10 were excluded. Values ≤5% were considered statistically significant. Data were stored and analyzed using the MedCalc^®^ package (Version 12.3.0 1993–2012 MedCalc Software bvba - MedCalc Software, Mariakerke, Belgium).

## III. RESULTS

A total of 87 subjects (4 males and 83 females) 70.1±4.9 aged, were examined. Mean BMI was 28.7±4.7(kg/m^2^): in particular 28 subjects (32.2%) resulted obese, 42 subjects (48.3%) resulted overweight, and only 17 subjects (19.5%) resulted with normal weight. Mean T score was - 1.2±1.2: in particular 13 subjects (14.9%) resulted osteoporotic; 43 subjects (49.5%) resulted osteopenic; and 31 subjects (35.6%) resulted with normal bone mineral density. Regarding adherence to MD, 9 subjects (10.3%) were poorly adherent; 41 subjects (47.2%) were average adherent; 37 subjects (42.5%) were highly adherent (score 0–5: low adherence; score 6–9: average adherence; score ≥10: high adherence). In Table 1 the socio-demographic, anthropometric measurement and metabolic characteristics of subjects have been shown.

**Fig. 1.**
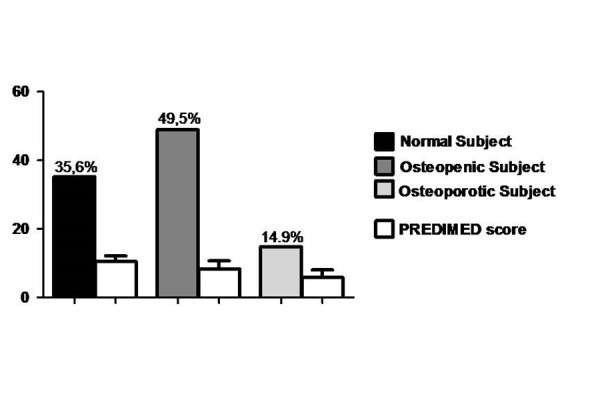
**Bone Health Status**

**Fig. 2.**
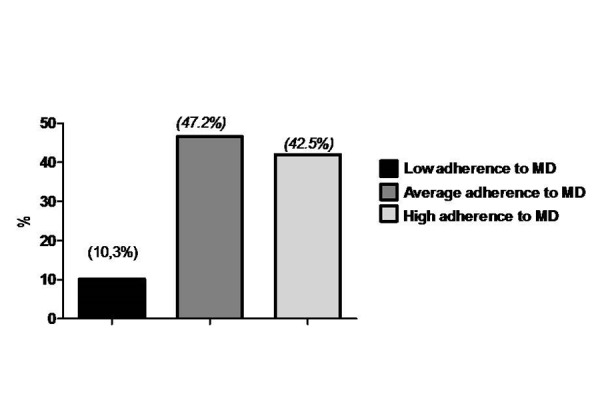
**Adherence to Mediterranean Diet (MD)**

In Table 2 the responses of each item included in PREDIMED questionnaire have been reported. The lowest consumption were represented by wine glasses, nuts and butter while, extra virgin olive oil (EVOO) was a major dietary components consumed. In Fig. 1 the percentages of subjects with osteopenia, osteoporosis and normal bone parameters have been shown. T-score was positively associated with PREDIMED score Osteoporotic subjects had the lowest PREDIMED score (5.8±2.2) Fig. 2.

## IV. DISCUSSION

Osteoporosis and osteoporosis-related fractures are growing problems within the aging population, and are associated with significant morbidity and mortality [6]. Osteoporosis is a bone disease characterized by an imbalance between bone resorption and formation: the rate of bone formation is often normal, whereas resorption by osteoclasts is increased, leading to a decrease in bone mass and microarchitectural alterations that results in bone fragility and increased fragility fractures’ risk [6]. Osteoporosis prevalence rises with advancing age, and in consideration of the demographic transition occurring worldwide it is projected to significantly increase in next future. In Italy, approximately 3.5 million persons are osteoporotic, with over 90.000 fractures yearly in >50 years aged or older population [7]. Almost 50% of women with hip osteoporotic fractures develop disability, with significant impact on independently living and, in most cases, institutionalization [7]. DXA is presently considered the gold standard imaging technique for the diagnosis of osteoporosis because it shows the best predictive value for fracture risk [7]. According to the World Health Organization (WHO), osteoporosis is defined as a bone mineral density (BMD) at the hip and/or the spine at least 2.5 standard deviations below the mean peak bone mass of young healthy adults as determined by dual-energy X-ray absorptiometry (DXA) [8].

Unfortunately, as other X-ray based techniques, DXA has specific limitations (e.g., use of ionizing radiation, large size of the equipment, high costs, limited availability) that limit its application for population screenings and primary care diagnosis. This has lead to an increasing interest in developing reliable pre-screening tools for osteoporosis such as quantitative ultrasound (QUS) scanners, which do not involve ionizing radiation exposure and represent a cheaper solution exploiting portable and widely available devices to screen osteoporosis in large population prevention projects, for an early diagnosis of this “silent disease”.

In humans, QUS indices were found to be associated with BMD as well, moreover QUS could predict risk for future fracture. In particular, the calcaneus has been chosen as a site for measurement since it is easily accessible, well-suited for optimizing the geometry of transmission of the US wave through it, and it contains approximately 90% trabecular bone which present a high metabolic turnover rate and a pattern of bone loss similar to the spine. Monitoring parameters derived are T-score, bone Mineral density (BMD) and QUI (Quantitative Ultrasound Index). On the other hand QUS is becoming a more and more reliable approach versus DEXA for assessing bone quality even if the US assessment of osteoporosis is currently used only as a pre-screening tool, requiring a subsequent diagnosis confirmation by means of a DXA evaluation [9,10].

A lot of risk factors have been identified for primary osteoporosis (e.g advancing age, female sex, white or asian race, low body weight / body mass index, family history of osteoporotic fractures, early menopause, sedentary lifestyle, excessive alcohol, caffeine, and tobacco use, low calcium and/or vitamin D intake, inadequate sun exposure [7].

Mediterranean diet (MD) represent a healthy eating pattern that is characterized by a high intake of fruit and vegetables, legumes, grains and cereals, fish and seafood and nuts; a low intake of dairy products, meat and meat products; and a moderate ethanol intake mainly in the form of wine and during meals [11]. EVOO is the main added lipid and its increased consumption is reflected in the high monounsaturated to saturated fatty acid intake [12]. In our study we investigated relationship between adherence to Mediterranean Diet and Bone health. T-score was calculated by calcaneal QUS; adherence to MD was assessed by PREDIMED Questionnaire. Our population included 87 subjects (4 males and 83 females); 13 subjects (14.9%) resulted osteoporotic; 43 (49.5%) resulted osteopenic; and 31 (35.6%) resulted normal for bone mineral density. Regarding adherence to MD, 9 subjects (10.3%) were poorly adherent; 41 (47.1%) were moderately adherent; 37 (42.5%) were highly adherent.

The interesting finding of our study is that T-score was positively associated with PREDIMED score, in particular the results of our study show that osteoporotic subjects have the lowest PREDIMED score (5.8±2.2), underlying that higher adherence to MD is associated with better bone health status. In the last two decades, several advancements have been realized, which warrant reconsideration of existing dietary strategies for osteoporosis prevention. To date, several studies have related additional nutrients with bone health, such as vitamins A, B, C, E, K; proteins; fats; minerals. It is now well known that an adequate nutrition is important in defining an optimal bone mass, as well as in preventing it [13].

Historically, calcium and vitamin D intake are necessary for good bone health; however, nutritional benefits to bone go beyond these two nutrients. A number of single food components have been suggested to play a role in osteoporosis prevention and several studies evaluated the role of singular nutrients on osteoporosis development [14] but our study, like other few studies, have gone beyond single nutrient associations and linked a dietary patterns (food groups) with bone health [15,16]. Regarding the possible linking between osteoporosis and MD the most likely hypothesis involves inflammatory mechanisms, since chronic inflammation has already been found to be associated with osteoporosis and aging-related bone loss [17,18]. It is well known that MD is associated with reduced risk for metabolic [19, 20], cardiovascular [21, 22], neoplastic [23] and other diseases [24–26], that has consistently been shown to provide a degree of protection against chronic degenerative diseases [27]. One of the most accredited explanation of this association is that the high content of different beneficial compounds, such as antioxidants and polyphenols, largely present in Mediterranean foods, such as plant foods, fruits and red wine, have anti-inflammatory properties. In particular, the monounsaturated fatty acids intake, whose major source is represented by EVOO, was found to be associated with a reduced prevalence of risk factors for major chronic inflammatory diseases [28]. Another significant aspect of this study is that PREDIMED Questionnaire, as well as as calcaneal QUS, represents a very reliable screening approach. PREDIMED appraises adherence to MD using only a brief 14-item questionnaire which is less time-demanding, less expensive and requires less collaboration from participants than the usual full-length food frequency questionnaire (FFQ) or other more comprehensive methods [29]. Moreover, this questionnaire allows to provide feedback to the participant immediately after the interview is completed. In fact, this 14-item tool is a key element in the intervention conducted in the PREDIMED trial and has been previously validated against the FFQ used in the study [30].

## V. CONCLUSION

These preliminary data show a significant correlation between adherence to Mediterranean Diet and bone health parameters such as T-score assessed by calcaneal QUS. The association between Mediterranean Diet and bone health highlights the potential beneficial effects of primary nutritional interventions promoting a Mediterranean food pattern rich in EVOO, fruits, vegetables, fish, chicken and whole grains, as a sustainable and safe adjuvant treatment in general population and in older adults in particular. Moreover, our study highlights the reliability of the assessment of bone impairment by calcaneal QUS to screen for and prevent frailty through community-based screening services, although we are aware that the reduced sample size and the validity of QUS in diagnosing osteoporosis might be a limitation of our study. Extension of the population sample size in the context of Perssilaa project will contribute to define the individual role of nutrients on bone health.

## Figures and Tables

**Table 1.** **Socio-demographic, anthropometric and metabolic characteristics of patients**

**Parameters**	**Patients (n=87)**
Age (years)	70.1±4.9
Height (m)	1.6±0.1
Weight (kg)	70.3±12.4
BMI (kg/m^2^)	28.7±4.7
SBP (mmHg)	139.7±19.0
DBP (mmHg)	81.5±9.2
WC (cm)	97.4±11.6
HC (cm)	108.3±10.0
WHR	0.9±0.1
T score	−1.2±1.2
BMD g/cm^2^	0.5±0.1

**Legend:** Results are expressed as mean±SD. ***BMI,*** body mass index; ***SBP***, systolic blood pressure; ***DBP***, diastolic blood pressure ***WC,*** Waist Circumference; ***HC***, hip circumference; ***WHR***, Waist to Hip Ratio; ***BMD,*** bone mineral density.

**Table 2.** **Response frequency of dietary components included in the PREDIMED questionnaire**

	**Questions**	**Patients**	
		**n**	**%**
1	Use of extra virgin olive oil as main culinary lipid	79	89.8
2	Extra virgin olive oil >4 tablespoons	61	69.3
3	Vegetables ≥2 servings/d	50	56.8
4	Fruits ≥3 servings/d	58	65.9
5	Red/processed meats <1/d	49	55.7
6	Butter, cream, margarine <1/d	47	53.4
7	Soda drinks <1/d	49	55.7
8	Wine glasses ≥7/wk	28	31.8
9	Legumes ≥3/we	60	68.2
10	Fish/seafood ≥3/we	65	73.9
11	Commercial sweets and confectionery ≤2/wk	50	56.8
12	Tree nuts ≥3/we	32	36.4
13	Poultry more than red meats	67	76.1
14	Use of sofrito sauce ≥2/we	64	72.7

**Legend:** The lowest consumption were represented by wine glasses, nuts and butter while, EVOO was a major dietary components consumed.

EVOO, Extra virgin olive oil; PREDIMED, PREvención con DIeta MEDiterránea.
